# Early detection of Varicella-Zoster Virus (VZV)-specific T-cells before seroconversion in primary varicella infection: case report

**DOI:** 10.1186/1743-422X-7-54

**Published:** 2010-03-06

**Authors:** Armin Baiker, Rudolf Haase, Josef Eberle, Maria Guadalupe Vizoso Pinto, Klaus-Ingmar Pfrepper, Andreas Petrich, Ludwig Deml, Hartmut Campe, Hans Nitschko, Gundula Jaeger

**Affiliations:** 1 Max von Pettenkofer-Institute, Ludwig-Maximilians-University, Munich, Germany; 2 Mikrogen GmbH, Neuried, Germany; 3 Lophius Biosciences GmbH, Regensburg, Germany; 4 Bavarian Health and Food Safety Authority (Bavarian LGL), Oberschleissheim, Germany

## Abstract

Here we report the case of a 54-year old, immunocompetent German patient with primary varicella whose Varicella-Zoster Virus (VZV)-specific T-cell responses could be detected early in infection and before the onset of seroconversion. This case demonstrates that the detection of VZV-specific T-cells may under certain circumstances support the diagnosis of a primary varicella infection, as for example in cases of atypical or subclinical varicella or in the absence of detectable VZV DNA in plasma.

## Background

Varicella-Zoster virus (VZV) causes varicella during primary infection and may cause herpes zoster after reactivation from latency. Varicella is typically diagnosed by characteristic clinical signs and usually does not require laboratory testing. Due to the introduction of mass vaccination programmes, however, the incidence of typical varicella has declined. This decline has led to a reduced experience of physicians in diagnosing varicella. Furthermore, an increased incidence of atypical and vaccination breakthrough varicella infections has been described [1-4]. For the early diagnosis of cases with severe or atypical varicella rapid VZV identification techniques are indicated to initiate specific antiviral therapy. Serological markers (i.e. VZV-IgM and/or VZV-IgG) are not appropriate for the laboratory diagnosis of early varicella, because they are detectable in a time-delayed manner [5]. Therefore, the method of choice for the rapid diagnosis of varicella is polymerase chain reaction (PCR) out of specimen collected from lesions [6]. Here we report that the rapid diagnosis of an early varicella infection may also be possible by the detection of VZV-specific CD4+ T-cells from peripheral blood.

## Case presentation

**Figure 1:**
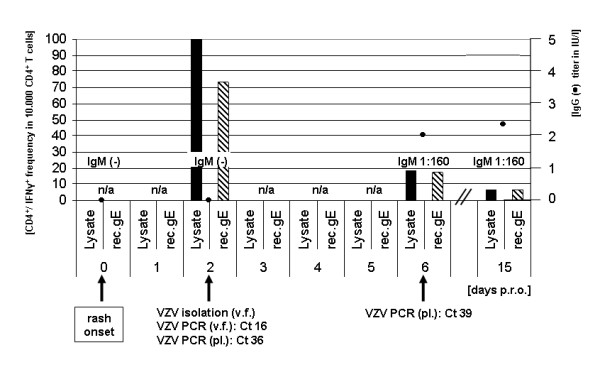
**Clinical and diagnostic parameters of the reported case**. A 54-year-old Caucasian presented with "varicella-like" skin rash and fever (day 0). Serological testing was negative for VZV-IgG (black circle) and VZV-IgM. A second blood sample was taken at day 2 p.r.o.. Serological testing for VZV-specific IgG (black circle) and IgM antibodies exhibited negative results. Abundant numbers of VZV-specific (CD4+/IFNγ+) T-cells could be detected within this second sample. Detected VZV-specific T-cell frequencies yielded 1% (100 in 10000 CD4+ T cells) when VZV lysate (Lysate) was used for stimulation (black bar), and 0.73% when utilizing recombinant glycoprotein E (rec.gE) (striped bar). Primary varicella could be diagnosed by virus isolation from vesicular fluid (v.f.) and by quantitative PCR for VZV-DNA out of v.f. or plasma (pl.) with Ct 16 and Ct 36, respectively. A third blood sample taken at day 6 p.r.o. demonstrated seroconversion with VZV-IgG titers of 2.2 IU/l (black circle) and VZV-IgM titers of 1:160. VZV-specific T-cells frequencies decreased to 0.19% when stimulating with Lysate and 0.17% when stimulating with rec.gE. Quantitative PCR for VZV-DNA out of plasma revealed Ct 39. A final blood sample taken at day 15 p.r.o. revealed VZV-IgG titers (black circle) of 2.4 IU/l and VZV-IgM titers of 1:160. VZV-specific T-cell frequencies further decreased to 0.063% when stimulating with Lysate, and 0.065% when stimulating with rec.gE. Abbreviations: not assayed (n/a), post rash onset (p.r.o.), vesicular fluid (v.f.), plasma (pl.), cycle threshold numbers (Ct), international units per liter as assayed by Siemens Enzygnost Anti-VZV/IgG (IU/l).

We report the case of a 54-year-old, immunocompetent German (Caucasian) man who presented with skin rash and fever (day 0). Due to the clinical appearance of a typical "varicella-like" rash phenotype, antiviral therapy with Brivudine was initiated at the day of the patient's presentation and rash onset. Serological analysis of blood taken at day 0 exhibited no VZV-specific antibodies indicative for a past (VZV-IgG) or primary (VZV-IgM) VZV infection. In order to confirm the suspicion of primary varicella, a second blood sample was taken at day 2 post rash onset (p.r.o.). Again, subsequent serological testing for VZV-specific IgG and IgM antibodies exhibited negative results. However, abundant numbers of VZV-specific CD4+ T-cells could be detected within this second sample. Analysis of VZV-specific T-cells was performed by a novel *in house *flow cytometry assay after intracellular interferon γ staining of isolated and *ex vivo *stimulated peripheral blood mononuclear cells (PBMCs). For a detailed protocol see additional file 1: PDF document describing our protocol for the detection of VZV-specific T-cells. Detected VZV-specific CD4+ T-cell titers yielded 1% when VZV lysate was used for stimulation, and 0.73% when utilizing recombinant glycoprotein E (gE). Apart from detecting abundant numbers of VZV-specific T-cells, primary varicella could also be diagnosed by virus isolation from vesicular fluid and by quantitative PCR for VZV-DNA out of vesicular fluid (Ct 16) or plasma (Ct 36). A third blood sample was taken at day 6 p.r.o.. Here, the respective VZV serology demonstrated seroconversion with VZV-IgG titers of 2.2 IU/l and VZV-IgM titers of 1:160. VZV-specific CD4+ T-cells titers decreased to 0.19% when stimulating with VZV lysate, and 0.17% when stimulating with recombinant gE. A quantitative PCR for VZV-DNA performed out of plasma was positive at low levels (Ct 39). A fourth and final blood sample was taken at day 15 p.r.o.. The respective VZV serology revealed VZV-IgG titers of 2.4 IU/l and VZV-IgM titers of 1:160. VZV-specific CD4+ T-cells titers further decreased to 0.063% when stimulating with VZV lysate, and 0.065% when stimulating with recombinant gE. VZV-DNA in plasma could not be detected any more by PCR at this stage. All relevant clinical and diagnostic parameters of the reported case are depicted in Figure 1 (Fig. 1).

## Conclusions

The development of novel assays for the detection of virus-specific T-cells will contribute to answer a variety of medically relevant questions that could so far not be addressed sufficiently. Among them (a) the diagnosis of a pathogen involvement if serological parameters are impaired, (b) the differentiation between different stages of viral infections, (c) the monitoring of the immune status of immunosuppressed patients after solid organ or bone marrow transplantation, (d) the examination of vaccine efficiency with respect to cellular immunity, or (e) the determination of immune correlates of protection towards a herpes zoster reactivation as indication for zoster vaccination. Here we report that the detection of VZV-specific T-cells may (f) additionally support the rapid diagnosis of an early varicella infection before seroconversion.

As to our knowledge, no systematic comparison between the onset kinetics of seroconversion and occurrence of VZV-specific T-cells within patients at early stages of varicella infection has been performed until today. Seroconversion has been described to appear one to seven days p.r.o. [7]. In contrast, activation of circulating T lymphocytes has been reported to occur earlier, in some patients already during the incubation period [7,8].

Within our patient, detectable VZV-specific CD4+/IFNγ+ T-cell titers were highest at day two p.r.o. and before the occurrence of seroconversion. At this time, titers of up to 1% of total CD4+ T-cells were detected. Such elevated CD4+ T-cell titers have been described during acute varicella infections [9]. The percentage of VZV-specific CD4+ T-cells is declining steadily over time, reaching levels of ~0.18% (± 0.01) at day six and ~0.064% (± 0.001) at day 15 p.r.o. (Fig. 1). The latter T-cell titers are slightly above those described for VZV-specific memory (<0.01%) [10]. No significant increase in IgG titers could be observed within our patient between day 6 (2.2 IU/l) and day 15 (2.4 IU/l), which could be explained by the early initiation of antiviral therapy at the day of rash onset.

We conclude that the analysis of VZV-specific CD4+ T-cells might support the rapid diagnosis of primary varicella under certain circumstances, as for example in cases of atypical or subclinical varicella or in the absence of detectable VZV DNA in plasma.

## Consent

Written informed consent was obtained from the patient for publication of this case report and any accompanying images. A copy of the written consent is available for review by the Editor-in-Chief of this journal.

## Competing interests

The authors declare that they have no competing interests.

## Authors' contributions

AB planned the project and wrote the paper. RH performed all FACS analyses for the detection of VZV-specific T cells. JE collected the patient samples. MGVP, K-IP and AP participated in the production of recombinant glycoprotein E for the *ex vivo *T cell stimulation. LD provided protocol and reagents for the urea treatment of VZV lysates for the *ex vivo *T-cell stimulation. HC provided the basic protocols for the analysis of virus-specific T-cells and helped interpreting the FACS data. HN performed the real time PCR analysis. GJ provided overall supervision of the project and helped with interpretation of clinical data. All authors read and approved the final manuscript.

## Supplementary Material

Additional file 1 **Protocol for the detection of VZV-specific T-cells**: This additional file provides a detailed protocol for the detection of VZV-specific T-cells from heparinized blood. Click here for file
