# Supplementary material for: Early detection of Varicella-Zoster Virus (VZV)-specific T-cells before seroconversion in primary varicella infection: case report
Source: Virol J. 2010 Mar 6;7:54. doi: 10.1186/1743-422X-7-54 (PMC2839977; doi:10.1186/1743-422X-7-54)
Supplement: Additional file 1 — Protocol for the detection of VZV-specific T-cells: This additional file provides a detailed protocol for the detection of VZV-specific T-cells from heparinized blood. [file 1743-422X-7-54-S1.PDF]

Additional file 1

Protocol for the detection of VZV-specific T-cells: This additional file provides a detailed protocol for the detection of VZV-specific T-cells from heparinized blood.

Blood sample handling and storage

Peripheral blood mononuclear cells (PBMCs) were isolated from heparinized patient blood samples at the day of blood collection using standard Ficoll-Isopaque density gradient centrifugation. PBMCs were resuspended in standard growth media supplemented with 10% DMSO and subsequently cryopreserved in 1ml aliquots (each aliquot corresponding to the number of PBMCs derived from 5ml heparinized blood) until enumeration of VZV-specific T cells was performed by intracellular cytokine staining (ICS) assays.

Preparation of VZV antigens

For the preparation of VZV lysate antigen, MRC5 cells (ATCC CCL-171) were infected with wildtype VZV and incubated until > 75% of all cells exhibited a cytopathic effect (CPE). The cells of one T75 flask were scraped off, pelleted and resuspended in 300µl urea buffer I (8M urea, 200mM NaCl, 20mM Tris, 2mM DTE, pH8.0). VZV lysates were incubated for 30min at room temperature and subsequently cleared by centrifugation at 10.000g. Cleared VZV lysates were aliquoted and stored at -20°C until further usage. Lysate prepared from uninfected MRC5 cells was used as control antigen.

Recombinant (bacterially expressed) VZV glycoprotein E (gE) antigen was derived from Mikrogen (Neuried, Germany) at a concentration of 0.64mg/ml in urea buffer II (8M urea, 20mM MES, 2mM DTE, 80mM NaCl, pH6.0). Urea buffer II (without any recombinant protein) was used as control antigen.

Ex vivo stimulation of PBMCs with VZV antigens

Aliquots of frozen PBMCs were thawed in a 37°C waterbath, washed once with 10ml and then resuspended in 2ml standard growth media. The 2ml PBMC suspension was divided

into two equal parts of 1ml, one being used for the VZV antigen stimulation the other being used for control stimulation. All stimulations were performed in a 1.5ml reaction volume consisting of 1000µl PBMC suspension, 493µl standard growth media, 2µl CD28Purified (#348040, BD Biosciences, Heidelberg, Germany), and 5µl antigen (or control antigen) solution. Stimulations were incubated at 37°C in a standard CO_2 incubator for a total of 7.5h, with 3µl Brefeldine A solution (#347688, BD Biosciences, Heidelberg, Germany) being added after 2.5 hours.

Intracellular cytokine staining (ICS)

After *ex vivo* stimulation, PBMCs were fixed and permeabilized using buffers and protocols for intracellular cytokine staining (BD Biosciences, Heidelberg, Germany). Intracellular IFNγ staining was performed using BD FastImmune™ Anti-IFN-γ/CD69/CD8/CD3FITC/PE/PerCP-Cy5.5/APC (#346048, BD Biosciences, Heidelberg, Germany) according to the manufacturers' instructions. Samples were analyzed on a BD FACS Canto II flow cytometer and CD4+ (defined as CD8- population) / IFNγ+ lymphocytes were enumerated using the BD FACS Diva software. Control stimulation results were subtracted from the VZV antigen stimulation results and the difference presented as CD4+/IFNγ+ T-cell frequency in 10000 CD4+ T cells. Results > 3 (= 0.03%) were considered positive.
